# Diaqua­[*N*,*N*′-bis­(2-pyridylmethyl­ene)propane-1,3-diamine]manganese(II) dibromide–aqua­bromido[*N*,*N*′-bis­(2-pyridylmethyl­ene)propane-1,3-diamine]manganese(II) bromide–dibromido[*N*,*N*′-bis­(2-pyridylmethyl­ene)propane-1,3-diamine]manganese(II) (1/2/1)

**DOI:** 10.1107/S1600536808041731

**Published:** 2008-12-13

**Authors:** In-Chul Hwang, Kwang Ha

**Affiliations:** a Department of Chemistry, Pohang University of Science and Technology, Pohang 790-784, Republic of Korea; b School of Applied Chemical Engineering, The Research Institute of Catalysis, Chonnam National University, Gwangju 500-757, Republic of Korea

## Abstract

There are three different Mn^II^ complexes in the asymmetric unit of the title compound, [Mn(C_15_H_16_N_4_)(H_2_O)_2_]Br_2_·2{[MnBr(C_15_H_16_N_4_)(H_2_O)]Br}·[MnBr_2_(C_15_H_16_N_4_)]. In the neutral complex, the Mn^2+^ ion is six-coordinated in a distorted octa­hedral environment by four N atoms of the tetra­dentate ligand *N*,*N*′-bis­(2-pyridylmethyl­ene)propane-1,3-diamine (bppd) and two bromide ligands. In the two cationic complexes, the Mn^2+^ ions are also six-coordinated in similar environments, but one Mn ion is coordinated by four N atoms of bppd, one Br atom and one O atom of a coordinating water mol­ecule, whereas the other Mn ion is coordinated by four N atoms of bppd and two O atoms of water ligands. The complexes with two coordinated Br atoms or two H_2_O ligands are disposed about a twofold axis through Mn and C atoms with the special positions (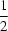, *y*, 0) and (0, *y*, 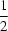), respectively. The compound displays inter­molecular O—H⋯Br hydrogen bonding. There are inter­molecular π–π inter­actions between adjacent pyridine rings, with centroid–centroid distances of 3.822 and 3.833 Å, and a C—H⋯O inter­action is also present.

## Related literature

For a structurally related complex, see: Hwang & Ha (2007 ▶). 
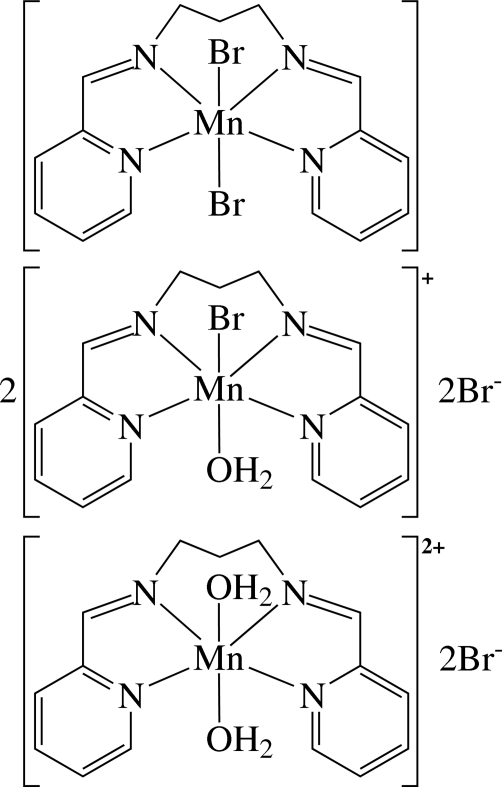


## Experimental

### 

#### Crystal data

[Mn(C_15_H_16_N_4_)(H_2_O)_2_]Br_2_·2{[MnBr(C_15_H_16_N_4_)(H_2_O)]Br}·[MnBr_2_(C_15_H_16_N_4_)]
*M* *_r_* = 1940.38
Monoclinic, 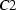

*a* = 28.559 (2) Å
*b* = 9.2318 (6) Å
*c* = 13.8990 (9) Å
β = 99.111 (2)°
*V* = 3618.2 (4) Å^3^
*Z* = 2
Mo *K*α radiation
μ = 5.16 mm^−1^
*T* = 293 (2) K
0.25 × 0.20 × 0.08 mm

#### Data collection

Bruker SMART 1000 CCD diffractometer
Absorption correction: multi-scan (*SADABS*; Bruker, 2000 ▶) *T*_min_ = 0.361, *T*_max_ = 0.662
14779 measured reflections
6854 independent reflections
5483 reflections with *I* > 2σ(*I*)
*R*_int_ = 0.035

#### Refinement

*R*[*F* ^2^ > 2σ(*F* ^2^)] = 0.045
*wR*(*F* ^2^) = 0.116
*S* = 0.98
6854 reflections
413 parameters
1 restraint
H-atom parameters constrained
Δρ_max_ = 1.30 e Å^−3^
Δρ_min_ = −2.37 e Å^−3^
Absolute structure: Flack (1983 ▶), 2901 Friedel pairs
Flack parameter: 0.06 (1)

### 

Data collection: *SMART* (Bruker, 2000 ▶); cell refinement: *SAINT* (Bruker, 2000 ▶); data reduction: *SAINT*; program(s) used to solve structure: *SHELXS97* (Sheldrick, 2008 ▶); program(s) used to refine structure: *SHELXL97* (Sheldrick, 2008 ▶); molecular graphics: *XP* (Siemens, 1990 ▶); software used to prepare material for publication: *SHELXL97*.

## Supplementary Material

Crystal structure: contains datablocks global, I. DOI: 10.1107/S1600536808041731/im2090sup1.cif

Structure factors: contains datablocks I. DOI: 10.1107/S1600536808041731/im2090Isup2.hkl

Additional supplementary materials:  crystallographic information; 3D view; checkCIF report

## Figures and Tables

**Table 1:** Hydrogen-bond geometry (Å, °)

*D*—H⋯*A*	*D*—H	H⋯*A*	*D*⋯*A*	*D*—H⋯*A*
O1—H1*WA*⋯Br2	0.932	2.34	3.261 (5)	170.1
O1—H1*WB*⋯Br2^i^	0.850	2.64	3.268 (5)	131.6
O2—H2*WA*⋯Br4	0.913	2.23	3.145 (5)	175.2
O2—H2*WB*⋯Br3^ii^	1.037	2.21	3.234 (4)	169.5
C4—H4⋯O2^i^	0.93	2.42	3.344 (9)	173
C29—H29⋯Br2^iii^	0.93	2.88	3.742 (7)	154

Symmetry codes: (i) 
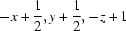
; (ii) 
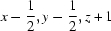
; (iii) 
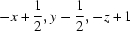
.

**Fig. 1.**
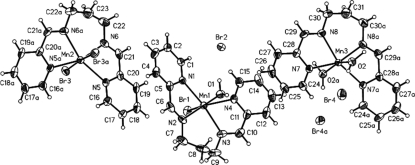
The structure of the title compound. Displacement ellipsoids are drawn at the 30% probability level.

**Fig. 2.**
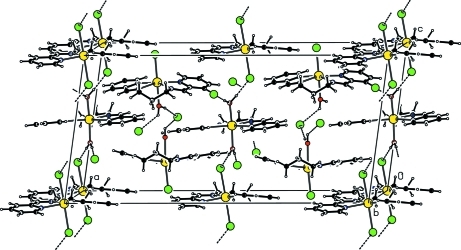
View of the unit-cell contents of the title compound. Hydrogen-bond interactions are drawn with dashed lines.

## supplementary crystallographic information

### Comment

The crystal structure of the title compound, [Mn(C_15_H_16_N_4_)(H_2_O)_2_]Br_2_[MnBr(C_15_H_16_N_4_)(H_2_O)]Br [MnBr_2_(C_15_H_16_N_4_)], contains three different Mn^II^ complexes in the unit cell (Fig. 1). In the neutral complex, the Mn^2+^ ion is six-coordinated in a distorted octahedral environment by four N atoms of the tetradentate ligand *N*,*N*'-bis-(pyridin-2-ylmethylene)-propane-1,3-diamine (bppd) occupying equatorial positions and two Br atoms occupying axial positions. The complex is disposed about a twofold axis passing through Mn2 and C23 which lie on the special positions (1/2, *y*, 0). Within the equatorial plane, the chelating angles lie in the range of 74.5 (2)°–87.9 (3)°. The apical Br3—Mn2—Br3a [Symmetry code: (*a*) 1 - *x*, *y*, -*z*] bond angle is 174.28 (7)°. In the two cationic complexes, the Mn^2+^ ions are also six-coordinated in distorted octahedral environments, but one Mn ion is coordinated by four N atoms of bppd, one Br atom and one O atom of a water ligand, whereas the other Mn ion is coordinated by four N atoms of bppd and two O atoms of water ligands. The cationic diaqua complex is disposed about a twofold axis passing through Mn3 and C31 which lie on the special positions (0, *y*, 1/2). Within the equatorial planes, the chelating angles lie in the range of 74.2 (2)°–85.9 (2)°. The apical Br1—Mn1—O1 and O2—Mn3—O2b [Symmetry code: (*b*) -*x*, *y*, 1 - *z*] bond angles are 167.08 (13)° and 160.2 (3)°, respectively. The compound displays intermolecular hydrogen bonds between the O atoms and the Br atoms (Fig. 2, Table 1). There are also intermolecular π-π interactions between adjacent pyridine rings, with centroid-to-centroid distances of 3.822 Å and 3.833 Å, and with dihedral angles between the ring planes of 9.8° and 4.4°.

### Experimental

A solution of MnBr_2_ (0.25 g, 1.16 mmol) and *N*,*N*'-bis-(pyridin-2-ylmethylene)-propane-1,3-diamine (0.30 g, 1.19 mmol) in EtOH (20 ml) was stirred for 1 h at room temparature. After adding diethyl ether to the solution, the formed precipitate was separated by filtration and washed with acetone and dried under vacuum, to give a dark yellow powder (0.50 g). Crystals suitable for X-ray analysis were obtained by slow evaporation from a methanolic solution. MS (FAB): m/*z* 386, 388 (Mn(bppd)Br^+^); IR (KBr): 3287 cm^-1^ (broad).

### Refinement

H atoms were positioned geometrically and allowed to ride on their respective parent atoms [C—H = 0.93 (aromatic) or 0.97 (CH_2_) Å and *U*_iso_(H) = 1.2*U*_eq_(C)]. The H atoms of the water ligands were located from Fourier difference maps, but not refined.  C23 and C31 which lie on special positions are highly disordered and were therfore refined with isotropic thermal parameters *U*_iso_ = 0.08 Å^2^.

### Crystal data

**Table:**

[Mn(C_15_H_16_N_4_)(H_2_O)_2_]Br_2_·2{[MnBr(C_15_H_16_N_4_)(H_2_O)]Br}·[MnBr_2_(C_15_H_16_N_4_)]	*F*(000) = 1912
*M**_r_* = 1940.38	*D*_x_ = 1.781 Mg m^−^^3^
Monoclinic, *C*2	Mo *K*α radiation, λ = 0.71073 Å
Hall symbol: C 2y	Cell parameters from 5299 reflections
*a* = 28.559 (2) Å	θ = 2.3–24.7°
*b* = 9.2318 (6) Å	µ = 5.16 mm^−^^1^
*c* = 13.8990 (9) Å	*T* = 293 K
β = 99.111 (2)°	Plate, yellow
*V* = 3618.2 (4) Å^3^	0.25 × 0.20 × 0.08 mm
*Z* = 2	

### Data collection

**Table:**

Bruker SMART 1000 CCD diffractometer	6854 independent reflections
Radiation source: fine-focus sealed tube	5483 reflections with *I* > 2σ(*I*)
graphite	*R*_int_ = 0.035
φ and ω scans	θ_max_ = 26.4°, θ_min_ = 1.4°
Absorption correction: multi-scan (*SADABS*; Bruker, 2000)	*h* = −32→35
*T*_min_ = 0.361, *T*_max_ = 0.662	*k* = −10→11
14779 measured reflections	*l* = −16→17

### Refinement

**Table:**

Refinement on *F*^2^	Secondary atom site location: difference Fourier map
Least-squares matrix: full	Hydrogen site location: inferred from neighbouring sites
*R*[*F*^2^ > 2σ(*F*^2^)] = 0.045	H-atom parameters constrained
*wR*(*F*^2^) = 0.116	*w* = 1/[σ^2^(*F*_o_^2^) + (0.0674*P*)^2^] where *P* = (*F*_o_^2^ + 2*F*_c_^2^)/3
*S* = 0.98	(Δ/σ)_max_ = 0.007
6854 reflections	Δρ_max_ = 1.30 e Å^−^^3^
413 parameters	Δρ_min_ = −2.37 e Å^−^^3^
1 restraint	Absolute structure: Flack (1983), 2901 Friedel pairs
Primary atom site location: structure-invariant direct methods	Flack parameter: 0.06 (1)

### Special details

**Table:**

Geometry. All e.s.d.'s (except the e.s.d. in the dihedral angle between two l.s. planes) are estimated using the full covariance matrix. The cell e.s.d.'s are taken into account individually in the estimation of e.s.d.'s in distances, angles and torsion angles; correlations between e.s.d.'s in cell parameters are only used when they are defined by crystal symmetry. An approximate (isotropic) treatment of cell e.s.d.'s is used for estimating e.s.d.'s involving l.s. planes.
Refinement. Refinement of *F*^2^ against ALL reflections. The weighted *R*-factor *wR* and goodness of fit *S* are based on *F*^2^, conventional *R*-factors *R* are based on *F*, with *F* set to zero for negative *F*^2^. The threshold expression of *F*^2^ > σ(*F*^2^) is used only for calculating *R*-factors(gt) *etc*. and is not relevant to the choice of reflections for refinement. *R*-factors based on *F*^2^ are statistically about twice as large as those based on *F*, and *R*- factors based on ALL data will be even larger.

### Fractional atomic coordinates and isotropic or equivalent isotropic displacement parameters (Å^2^)

**Table:**

	*x*	*y*	*z*	*U*_iso_*/*U*_eq_	Occ. (<1)
Mn1	0.23354 (3)	0.67117 (10)	0.21033 (6)	0.0378 (2)	
Br1	0.21279 (2)	0.65427 (9)	0.02208 (4)	0.0549 (2)	
Br2	0.29708 (2)	0.33983 (8)	0.47346 (5)	0.05364 (19)	
O1	0.24730 (16)	0.6324 (5)	0.3731 (3)	0.0525 (12)	
H1WA	0.2627	0.5465	0.3944	0.22 (7)*	
H1WB	0.2500	0.6545	0.4331	0.17 (5)*	
N1	0.30105 (16)	0.5427 (6)	0.2189 (3)	0.0385 (11)	
N2	0.2915 (2)	0.8352 (6)	0.2394 (4)	0.0481 (13)	
N3	0.1850 (2)	0.8506 (7)	0.2407 (4)	0.0519 (14)	
N4	0.16399 (18)	0.5636 (7)	0.2259 (4)	0.0448 (13)	
C1	0.3072 (2)	0.4038 (8)	0.2018 (5)	0.0508 (17)	
H1	0.2804	0.3481	0.1808	0.061*	
C2	0.3508 (3)	0.3360 (10)	0.2131 (5)	0.0605 (19)	
H2	0.3535	0.2376	0.2006	0.073*	
C3	0.3906 (3)	0.4207 (11)	0.2441 (5)	0.065 (2)	
H3	0.4207	0.3794	0.2530	0.078*	
C4	0.3853 (2)	0.5630 (10)	0.2611 (5)	0.060 (2)	
H4	0.4117	0.6200	0.2826	0.072*	
C5	0.3401 (2)	0.6250 (8)	0.2463 (4)	0.0493 (17)	
C6	0.3324 (3)	0.7804 (8)	0.2573 (5)	0.054 (2)	
H6	0.3583	0.8401	0.2779	0.065*	
C7	0.2857 (3)	0.9905 (8)	0.2476 (5)	0.068 (2)	
H7A	0.2797	1.0323	0.1827	0.081*	
H7B	0.3149	1.0317	0.2815	0.081*	
C8	0.2468 (3)	1.0300 (9)	0.2995 (6)	0.083 (3)	
H8A	0.2501	1.1317	0.3167	0.099*	
H8B	0.2505	0.9755	0.3599	0.099*	
C9	0.1979 (3)	1.0063 (9)	0.2477 (6)	0.078 (3)	
H9A	0.1951	1.0466	0.1825	0.094*	
H9B	0.1757	1.0574	0.2817	0.094*	
C10	0.1444 (3)	0.8098 (10)	0.2559 (5)	0.060 (2)	
H10	0.1231	0.8777	0.2731	0.072*	
C11	0.1306 (2)	0.6568 (10)	0.2468 (4)	0.0523 (18)	
C12	0.0858 (3)	0.6059 (13)	0.2581 (5)	0.073 (3)	
H12	0.0632	0.6699	0.2745	0.087*	
C13	0.0751 (3)	0.4644 (13)	0.2454 (6)	0.075 (3)	
H13	0.0450	0.4310	0.2511	0.090*	
C14	0.1082 (3)	0.3719 (12)	0.2245 (6)	0.075 (3)	
H14	0.1016	0.2737	0.2162	0.090*	
C15	0.1534 (3)	0.4265 (9)	0.2153 (5)	0.059 (2)	
H15	0.1765	0.3625	0.2011	0.070*	
Mn2	0.5000	0.51666 (15)	0.0000	0.0429 (3)	
Br3	0.47098 (2)	0.53137 (8)	−0.19582 (5)	0.0545 (2)	
N5	0.43259 (17)	0.6317 (6)	0.0202 (4)	0.0448 (13)	
N6	0.4462 (2)	0.3436 (6)	0.0014 (3)	0.0492 (13)	
C16	0.4249 (2)	0.7700 (8)	0.0343 (5)	0.0545 (18)	
H16	0.4510	0.8315	0.0457	0.065*	
C17	0.3804 (3)	0.8294 (10)	0.0331 (5)	0.064 (2)	
H17	0.3770	0.9284	0.0423	0.077*	
C18	0.3419 (3)	0.7424 (11)	0.0184 (5)	0.063 (2)	
H18	0.3116	0.7806	0.0164	0.075*	
C19	0.3483 (2)	0.5952 (10)	0.0063 (5)	0.0551 (19)	
H19	0.3226	0.5321	−0.0024	0.066*	
C20	0.3946 (2)	0.5435 (9)	0.0075 (4)	0.0490 (17)	
C21	0.4047 (3)	0.3865 (9)	0.0003 (5)	0.0542 (19)	
H21	0.3800	0.3199	−0.0051	0.065*	
C22	0.4544 (3)	0.1870 (9)	−0.0035 (6)	0.070 (2)	
H22A	0.4430	0.1393	0.0506	0.084*	
H22B	0.4369	0.1486	−0.0636	0.084*	
C23	0.5000	0.1605 (15)	0.0000	0.080*	
H23A	0.5033	0.0954	0.0555	0.096*	0.50
H23B	0.4967	0.0954	−0.0555	0.096*	0.50
Mn3	0.0000	0.20194 (14)	0.5000	0.0406 (3)	
Br4	0.05416 (3)	0.54204 (10)	0.73983 (7)	0.0786 (3)	
O2	0.01481 (15)	0.2422 (5)	0.6552 (3)	0.0505 (11)	
H2WA	0.0279	0.3279	0.6786	0.044 (17)*	
H2WB	0.0013	0.1642	0.6970	0.045 (17)*	
N7	0.07095 (18)	0.3114 (6)	0.4971 (4)	0.0422 (13)	
N8	0.0515 (2)	0.0231 (7)	0.4885 (4)	0.0524 (14)	
C24	0.0825 (3)	0.4523 (9)	0.5045 (5)	0.0551 (19)	
H24	0.0589	0.5179	0.5141	0.066*	
C25	0.1267 (3)	0.5054 (10)	0.4988 (5)	0.061 (2)	
H25	0.1332	0.6040	0.5048	0.073*	
C26	0.1613 (3)	0.4073 (13)	0.4840 (5)	0.072 (3)	
H26	0.1916	0.4396	0.4786	0.087*	
C27	0.1512 (2)	0.2644 (11)	0.4771 (5)	0.060 (2)	
H27	0.1745	0.1974	0.4687	0.071*	
C28	0.1051 (2)	0.2195 (8)	0.4830 (5)	0.0470 (16)	
C29	0.0923 (3)	0.0629 (9)	0.4800 (5)	0.059 (2)	
H29	0.1150	−0.0060	0.4715	0.071*	
C30	0.0429 (3)	−0.1332 (9)	0.4859 (7)	0.085 (3)	
H30A	0.0481	−0.1700	0.4231	0.102*	
H30B	0.0659	−0.1789	0.5353	0.102*	
C31	0.0000	−0.1720 (16)	0.5000	0.080*	
H31A	0.0083	−0.2366	0.5551	0.096*	0.50
H31B	−0.0083	−0.2366	0.4449	0.096*	0.50

### Atomic displacement parameters (Å^2^)

**Table:**

	*U*^11^	*U*^22^	*U*^33^	*U*^12^	*U*^13^	*U*^23^
Mn1	0.0336 (5)	0.0370 (6)	0.0422 (5)	−0.0022 (4)	0.0043 (4)	−0.0030 (4)
Br1	0.0469 (4)	0.0742 (5)	0.0422 (3)	0.0010 (4)	0.0025 (3)	−0.0013 (3)
Br2	0.0557 (4)	0.0429 (4)	0.0644 (4)	0.0008 (3)	0.0158 (3)	0.0081 (3)
O1	0.067 (3)	0.053 (3)	0.038 (2)	0.011 (3)	0.009 (2)	0.002 (2)
N1	0.034 (3)	0.043 (3)	0.039 (2)	−0.004 (2)	0.005 (2)	0.002 (2)
N2	0.065 (4)	0.038 (3)	0.040 (3)	−0.016 (3)	0.005 (3)	0.000 (2)
N3	0.061 (4)	0.043 (3)	0.048 (3)	0.015 (3)	−0.005 (3)	−0.005 (3)
N4	0.037 (3)	0.055 (4)	0.043 (3)	−0.007 (3)	0.007 (2)	−0.003 (3)
C1	0.046 (4)	0.051 (4)	0.058 (4)	0.000 (3)	0.017 (3)	0.012 (3)
C2	0.062 (5)	0.058 (5)	0.067 (5)	0.010 (4)	0.027 (4)	0.012 (4)
C3	0.045 (4)	0.099 (7)	0.052 (4)	0.024 (4)	0.011 (3)	0.006 (4)
C4	0.037 (4)	0.093 (7)	0.050 (4)	0.000 (4)	0.006 (3)	−0.001 (4)
C5	0.038 (4)	0.069 (5)	0.041 (3)	−0.010 (3)	0.008 (3)	−0.001 (3)
C6	0.058 (5)	0.063 (5)	0.040 (4)	−0.035 (4)	0.004 (3)	−0.007 (3)
C7	0.105 (7)	0.043 (4)	0.053 (4)	−0.027 (4)	0.005 (4)	−0.009 (3)
C8	0.133 (8)	0.032 (4)	0.073 (5)	−0.005 (5)	−0.012 (5)	−0.006 (4)
C9	0.107 (7)	0.040 (5)	0.080 (5)	0.019 (5)	−0.009 (5)	−0.006 (4)
C10	0.059 (5)	0.071 (6)	0.049 (4)	0.016 (4)	0.005 (3)	−0.008 (4)
C11	0.039 (4)	0.079 (6)	0.038 (3)	0.011 (4)	0.003 (3)	0.004 (4)
C12	0.043 (4)	0.125 (9)	0.051 (4)	0.010 (5)	0.012 (3)	0.011 (5)
C13	0.047 (5)	0.122 (9)	0.057 (5)	−0.027 (5)	0.006 (4)	0.011 (5)
C14	0.063 (5)	0.101 (8)	0.062 (5)	−0.036 (5)	0.010 (4)	0.007 (5)
C15	0.054 (4)	0.064 (5)	0.059 (4)	−0.017 (4)	0.013 (3)	−0.006 (4)
Mn2	0.0395 (7)	0.0358 (8)	0.0548 (8)	0.000	0.0117 (6)	0.000
Br3	0.0571 (4)	0.0607 (5)	0.0463 (3)	−0.0058 (4)	0.0098 (3)	0.0110 (3)
N5	0.031 (3)	0.050 (4)	0.054 (3)	−0.005 (2)	0.011 (2)	0.002 (3)
N6	0.067 (4)	0.043 (3)	0.037 (3)	−0.011 (3)	0.006 (2)	0.003 (2)
C16	0.044 (4)	0.060 (5)	0.060 (4)	0.002 (3)	0.009 (3)	−0.005 (3)
C17	0.051 (4)	0.068 (5)	0.077 (5)	0.017 (4)	0.024 (4)	0.005 (4)
C18	0.040 (4)	0.102 (7)	0.048 (4)	0.004 (4)	0.013 (3)	0.011 (4)
C19	0.033 (4)	0.091 (6)	0.043 (4)	−0.007 (4)	0.013 (3)	0.005 (3)
C20	0.040 (4)	0.066 (5)	0.042 (3)	−0.009 (3)	0.009 (3)	0.010 (3)
C21	0.047 (4)	0.071 (6)	0.046 (4)	−0.026 (4)	0.009 (3)	0.001 (3)
C22	0.079 (5)	0.050 (5)	0.075 (5)	−0.014 (4)	−0.008 (4)	0.004 (4)
Mn3	0.0376 (7)	0.0312 (8)	0.0555 (8)	0.000	0.0152 (6)	0.000
Br4	0.0802 (6)	0.0722 (6)	0.0899 (6)	−0.0243 (5)	0.0334 (5)	−0.0249 (5)
O2	0.051 (3)	0.050 (3)	0.051 (3)	−0.002 (2)	0.012 (2)	0.002 (2)
N7	0.035 (3)	0.046 (4)	0.046 (3)	−0.002 (3)	0.010 (2)	0.001 (2)
N8	0.062 (4)	0.042 (3)	0.056 (3)	0.010 (3)	0.018 (3)	−0.001 (3)
C24	0.055 (4)	0.054 (5)	0.058 (4)	−0.012 (4)	0.012 (3)	0.001 (3)
C25	0.048 (4)	0.068 (6)	0.063 (4)	−0.026 (4)	−0.001 (3)	0.006 (4)
C26	0.045 (5)	0.124 (9)	0.047 (4)	−0.024 (5)	0.001 (3)	0.014 (5)
C27	0.034 (4)	0.087 (6)	0.057 (4)	0.003 (4)	0.006 (3)	0.007 (4)
C28	0.039 (4)	0.060 (5)	0.043 (3)	0.005 (3)	0.007 (3)	−0.005 (3)
C29	0.057 (5)	0.060 (5)	0.060 (4)	0.027 (4)	0.011 (4)	0.003 (4)
C30	0.102 (7)	0.043 (5)	0.122 (7)	0.015 (5)	0.056 (6)	−0.003 (5)

### Geometric parameters (Å, °)

**Table:**

Mn1—N2	2.232 (6)	Mn2—Br3	2.7200 (7)
Mn1—N3	2.244 (6)	N5—C16	1.315 (9)
Mn1—N1	2.250 (5)	N5—C20	1.345 (8)
Mn1—N4	2.262 (5)	N6—C21	1.247 (9)
Mn1—O1	2.263 (4)	N6—C22	1.468 (10)
Mn1—Br1	2.5944 (10)	C16—C17	1.382 (10)
O1—H1WA	0.932	C16—H16	0.9300
O1—H1WB	0.850	C17—C18	1.352 (11)
N1—C1	1.321 (9)	C17—H17	0.9300
N1—C5	1.352 (8)	C18—C19	1.385 (11)
N2—C6	1.263 (9)	C18—H18	0.9300
N2—C7	1.450 (9)	C19—C20	1.403 (9)
N3—C10	1.268 (9)	C19—H19	0.9300
N3—C9	1.483 (10)	C20—C21	1.484 (11)
N4—C15	1.303 (9)	C21—H21	0.9300
N4—C11	1.349 (9)	C22—C23	1.319 (8)
C1—C2	1.382 (10)	C22—H22A	0.9700
C1—H1	0.9300	C22—H22B	0.9700
C2—C3	1.390 (11)	C23—C22^i^	1.319 (8)
C2—H2	0.9300	C23—H23A	0.9700
C3—C4	1.347 (12)	C23—H23B	0.9700
C3—H3	0.9300	Mn3—O2	2.164 (4)
C4—C5	1.398 (10)	Mn3—O2^ii^	2.164 (4)
C4—H4	0.9300	Mn3—N8^ii^	2.234 (6)
C5—C6	1.462 (11)	Mn3—N8	2.234 (6)
C6—H6	0.9300	Mn3—N7	2.270 (5)
C7—C8	1.464 (11)	Mn3—N7^ii^	2.270 (5)
C7—H7A	0.9700	O2—H2WA	0.913
C7—H7B	0.9700	O2—H2WB	1.037
C8—C9	1.483 (11)	N7—C28	1.330 (8)
C8—H8A	0.9700	N7—C24	1.341 (9)
C8—H8B	0.9700	N8—C29	1.244 (9)
C9—H9A	0.9700	N8—C30	1.463 (10)
C9—H9B	0.9700	C24—C25	1.370 (10)
C10—C11	1.467 (12)	C24—H24	0.9300
C10—H10	0.9300	C25—C26	1.378 (12)
C11—C12	1.397 (11)	C25—H25	0.9300
C12—C13	1.347 (13)	C26—C27	1.350 (11)
C12—H12	0.9300	C26—H26	0.9300
C13—C14	1.339 (13)	C27—C28	1.394 (10)
C13—H13	0.9300	C27—H27	0.9300
C14—C15	1.410 (10)	C28—C29	1.491 (11)
C14—H14	0.9300	C29—H29	0.9300
C15—H15	0.9300	C30—C31	1.319 (9)
Mn2—N6	2.219 (6)	C30—H30A	0.9700
Mn2—N6^i^	2.219 (6)	C30—H30B	0.9700
Mn2—N5	2.255 (5)	C31—C30^ii^	1.319 (9)
Mn2—N5^i^	2.255 (5)	C31—H31A	0.9700
Mn2—Br3^i^	2.7200 (7)	C31—H31B	0.9700
			
N2—Mn1—N3	85.9 (2)	N6^i^—Mn2—Br3	97.41 (13)
N2—Mn1—N1	75.1 (2)	N5—Mn2—Br3	88.29 (13)
N3—Mn1—N1	158.3 (2)	N5^i^—Mn2—Br3	89.01 (13)
N2—Mn1—N4	156.6 (2)	Br3^i^—Mn2—Br3	174.28 (7)
N3—Mn1—N4	74.2 (2)	C16—N5—C20	117.2 (6)
N1—Mn1—N4	121.4 (2)	C16—N5—Mn2	129.9 (5)
N2—Mn1—O1	85.40 (18)	C20—N5—Mn2	112.6 (5)
N3—Mn1—O1	86.76 (19)	C21—N6—C22	118.1 (6)
N1—Mn1—O1	81.49 (17)	C21—N6—Mn2	115.4 (5)
N4—Mn1—O1	81.42 (17)	C22—N6—Mn2	126.4 (5)
N2—Mn1—Br1	105.54 (13)	N5—C16—C17	123.9 (7)
N3—Mn1—Br1	100.65 (13)	N5—C16—H16	118.0
N1—Mn1—Br1	94.51 (12)	C17—C16—H16	118.0
N4—Mn1—Br1	90.37 (13)	C18—C17—C16	119.4 (8)
O1—Mn1—Br1	167.08 (13)	C18—C17—H17	120.3
Mn1—O1—H1WA	116.8	C16—C17—H17	120.3
Mn1—O1—H1WB	156.5	C17—C18—C19	118.7 (8)
H1WA—O1—H1WB	85.9	C17—C18—H18	120.7
C1—N1—C5	118.0 (6)	C19—C18—H18	120.7
C1—N1—Mn1	129.7 (4)	C18—C19—C20	118.4 (7)
C5—N1—Mn1	112.3 (5)	C18—C19—H19	120.8
C6—N2—C7	119.6 (6)	C20—C19—H19	120.8
C6—N2—Mn1	113.6 (5)	N5—C20—C19	122.3 (7)
C7—N2—Mn1	126.5 (5)	N5—C20—C21	116.1 (6)
C10—N3—C9	120.1 (7)	C19—C20—C21	121.6 (7)
C10—N3—Mn1	114.9 (5)	N6—C21—C20	120.2 (6)
C9—N3—Mn1	125.0 (5)	N6—C21—H21	119.9
C15—N4—C11	119.0 (7)	C20—C21—H21	119.9
C15—N4—Mn1	127.3 (5)	C23—C22—N6	110.2 (9)
C11—N4—Mn1	113.7 (5)	C23—C22—H22A	109.6
N1—C1—C2	124.2 (7)	N6—C22—H22A	109.6
N1—C1—H1	117.9	C23—C22—H22B	109.6
C2—C1—H1	117.9	N6—C22—H22B	109.6
C1—C2—C3	117.3 (8)	H22A—C22—H22B	108.1
C1—C2—H2	121.4	C22^i^—C23—C22	158.6 (14)
C3—C2—H2	121.4	C22^i^—C23—H23A	96.6
C4—C3—C2	119.6 (7)	C22—C23—H23A	96.6
C4—C3—H3	120.2	C22^i^—C23—H23B	96.6
C2—C3—H3	120.2	C22—C23—H23B	96.6
C3—C4—C5	120.1 (8)	H23A—C23—H23B	103.5
C3—C4—H4	120.0	O2—Mn3—O2^ii^	160.2 (3)
C5—C4—H4	120.0	O2—Mn3—N8^ii^	94.74 (19)
N1—C5—C4	120.8 (7)	O2^ii^—Mn3—N8^ii^	99.85 (19)
N1—C5—C6	116.8 (6)	O2—Mn3—N8	99.85 (19)
C4—C5—C6	122.4 (7)	O2^ii^—Mn3—N8	94.74 (19)
N2—C6—C5	121.4 (6)	N8^ii^—Mn3—N8	84.7 (3)
N2—C6—H6	119.3	O2—Mn3—N7	84.67 (17)
C5—C6—H6	119.3	O2^ii^—Mn3—N7	86.56 (17)
N2—C7—C8	112.9 (6)	N8^ii^—Mn3—N7	158.4 (2)
N2—C7—H7A	109.0	N8—Mn3—N7	74.2 (2)
C8—C7—H7A	109.0	O2—Mn3—N7^ii^	86.56 (17)
N2—C7—H7B	109.0	O2^ii^—Mn3—N7^ii^	84.67 (17)
C8—C7—H7B	109.0	N8^ii^—Mn3—N7^ii^	74.2 (2)
H7A—C7—H7B	107.8	N8—Mn3—N7^ii^	158.4 (2)
C7—C8—C9	116.9 (7)	N7—Mn3—N7^ii^	127.1 (3)
C7—C8—H8A	108.1	Mn3—O2—H2WA	120.5
C9—C8—H8A	108.1	Mn3—O2—H2WB	114.1
C7—C8—H8B	108.1	H2WA—O2—H2WB	124.8
C9—C8—H8B	108.1	C28—N7—C24	116.9 (6)
H8A—C8—H8B	107.3	C28—N7—Mn3	113.3 (5)
C8—C9—N3	112.3 (7)	C24—N7—Mn3	129.7 (5)
C8—C9—H9A	109.1	C29—N8—C30	116.5 (7)
N3—C9—H9A	109.1	C29—N8—Mn3	115.2 (5)
C8—C9—H9B	109.1	C30—N8—Mn3	128.3 (5)
N3—C9—H9B	109.1	N7—C24—C25	124.0 (8)
H9A—C9—H9B	107.9	N7—C24—H24	118.0
N3—C10—C11	120.7 (7)	C25—C24—H24	118.0
N3—C10—H10	119.6	C24—C25—C26	117.5 (8)
C11—C10—H10	119.6	C24—C25—H25	121.2
N4—C11—C12	120.1 (9)	C26—C25—H25	121.2
N4—C11—C10	116.4 (6)	C27—C26—C25	120.1 (8)
C12—C11—C10	123.5 (8)	C27—C26—H26	119.9
C13—C12—C11	120.2 (9)	C25—C26—H26	119.9
C13—C12—H12	119.9	C26—C27—C28	118.6 (8)
C11—C12—H12	119.9	C26—C27—H27	120.7
C14—C13—C12	119.6 (8)	C28—C27—H27	120.7
C14—C13—H13	120.2	N7—C28—C27	122.7 (7)
C12—C13—H13	120.2	N7—C28—C29	116.1 (6)
C13—C14—C15	118.7 (9)	C27—C28—C29	121.1 (7)
C13—C14—H14	120.7	N8—C29—C28	120.9 (7)
C15—C14—H14	120.7	N8—C29—H29	119.6
N4—C15—C14	122.4 (8)	C28—C29—H29	119.6
N4—C15—H15	118.8	C31—C30—N8	114.9 (9)
C14—C15—H15	118.8	C31—C30—H30A	108.6
N6—Mn2—N6^i^	87.9 (3)	N8—C30—H30A	108.6
N6—Mn2—N5	74.5 (2)	C31—C30—H30B	108.6
N6^i^—Mn2—N5	161.2 (2)	N8—C30—H30B	108.6
N6—Mn2—N5^i^	161.2 (2)	H30A—C30—H30B	107.5
N6^i^—Mn2—N5^i^	74.5 (2)	C30—C31—C30^ii^	148.5 (14)
N5—Mn2—N5^i^	123.8 (3)	C30—C31—H31A	99.6
N6—Mn2—Br3^i^	97.41 (13)	C30^ii^—C31—H31A	99.6
N6^i^—Mn2—Br3^i^	86.73 (13)	C30—C31—H31B	99.6
N5—Mn2—Br3^i^	89.01 (13)	C30^ii^—C31—H31B	99.6
N5^i^—Mn2—Br3^i^	88.29 (13)	H31A—C31—H31B	104.1
N6—Mn2—Br3	86.73 (13)		
			
N2—Mn1—N1—C1	−174.0 (6)	N6—Mn2—N5—C16	176.3 (6)
N3—Mn1—N1—C1	156.5 (6)	N6^i^—Mn2—N5—C16	155.2 (6)
N4—Mn1—N1—C1	24.1 (6)	N5^i^—Mn2—N5—C16	−8.9 (6)
O1—Mn1—N1—C1	98.6 (5)	Br3^i^—Mn2—N5—C16	78.4 (6)
Br1—Mn1—N1—C1	−69.1 (5)	Br3—Mn2—N5—C16	−96.6 (6)
N2—Mn1—N1—C5	6.4 (4)	N6—Mn2—N5—C20	−9.6 (4)
N3—Mn1—N1—C5	−23.2 (7)	N6^i^—Mn2—N5—C20	−30.7 (8)
N4—Mn1—N1—C5	−155.5 (4)	N5^i^—Mn2—N5—C20	165.1 (5)
O1—Mn1—N1—C5	−81.1 (4)	Br3^i^—Mn2—N5—C20	−107.6 (4)
Br1—Mn1—N1—C5	111.3 (4)	Br3—Mn2—N5—C20	77.4 (4)
N3—Mn1—N2—C6	162.0 (5)	N6^i^—Mn2—N6—C21	−178.5 (6)
N1—Mn1—N2—C6	−7.5 (4)	N5—Mn2—N6—C21	8.2 (5)
N4—Mn1—N2—C6	130.7 (6)	N5^i^—Mn2—N6—C21	−158.2 (6)
O1—Mn1—N2—C6	74.9 (5)	Br3^i^—Mn2—N6—C21	95.1 (5)
Br1—Mn1—N2—C6	−98.1 (4)	Br3—Mn2—N6—C21	−80.9 (5)
N3—Mn1—N2—C7	−12.3 (5)	N6^i^—Mn2—N6—C22	−3.1 (4)
N1—Mn1—N2—C7	178.3 (6)	N5—Mn2—N6—C22	−176.5 (6)
N4—Mn1—N2—C7	−43.5 (8)	N5^i^—Mn2—N6—C22	17.2 (9)
O1—Mn1—N2—C7	−99.3 (5)	Br3^i^—Mn2—N6—C22	−89.6 (5)
Br1—Mn1—N2—C7	87.7 (5)	Br3—Mn2—N6—C22	94.4 (5)
N2—Mn1—N3—C10	−164.7 (5)	C20—N5—C16—C17	−2.5 (10)
N1—Mn1—N3—C10	−136.1 (6)	Mn2—N5—C16—C17	171.3 (5)
N4—Mn1—N3—C10	2.9 (5)	N5—C16—C17—C18	1.2 (11)
O1—Mn1—N3—C10	−79.1 (5)	C16—C17—C18—C19	0.9 (11)
Br1—Mn1—N3—C10	90.3 (5)	C17—C18—C19—C20	−1.5 (11)
N2—Mn1—N3—C9	12.7 (6)	C16—N5—C20—C19	1.8 (9)
N1—Mn1—N3—C9	41.3 (9)	Mn2—N5—C20—C19	−173.0 (5)
N4—Mn1—N3—C9	−179.7 (6)	C16—N5—C20—C21	−174.9 (6)
O1—Mn1—N3—C9	98.3 (6)	Mn2—N5—C20—C21	10.2 (7)
Br1—Mn1—N3—C9	−92.3 (5)	C18—C19—C20—N5	0.2 (10)
N2—Mn1—N4—C15	−150.2 (6)	C18—C19—C20—C21	176.7 (6)
N3—Mn1—N4—C15	177.3 (6)	C22—N6—C21—C20	178.5 (6)
N1—Mn1—N4—C15	−19.2 (7)	Mn2—N6—C21—C20	−5.7 (8)
O1—Mn1—N4—C15	−93.7 (6)	N5—C20—C21—N6	−3.4 (9)
Br1—Mn1—N4—C15	76.3 (6)	C19—C20—C21—N6	179.9 (6)
N2—Mn1—N4—C11	31.2 (8)	C21—N6—C22—C23	−179.0 (5)
N3—Mn1—N4—C11	−1.4 (4)	Mn2—N6—C22—C23	5.8 (8)
N1—Mn1—N4—C11	162.1 (4)	N6—C22—C23—C22^i^	−3.2 (5)
O1—Mn1—N4—C11	87.7 (4)	O2—Mn3—N7—C28	106.6 (4)
Br1—Mn1—N4—C11	−102.4 (4)	O2^ii^—Mn3—N7—C28	−91.2 (4)
C5—N1—C1—C2	2.2 (9)	N8^ii^—Mn3—N7—C28	17.2 (8)
Mn1—N1—C1—C2	−177.5 (5)	N8—Mn3—N7—C28	4.8 (4)
N1—C1—C2—C3	−0.3 (10)	N7^ii^—Mn3—N7—C28	−171.8 (5)
C1—C2—C3—C4	−0.3 (10)	O2—Mn3—N7—C24	−75.6 (6)
C2—C3—C4—C5	−0.9 (11)	O2^ii^—Mn3—N7—C24	86.7 (6)
C1—N1—C5—C4	−3.3 (8)	N8^ii^—Mn3—N7—C24	−165.0 (6)
Mn1—N1—C5—C4	176.4 (5)	N8—Mn3—N7—C24	−177.4 (6)
C1—N1—C5—C6	175.4 (6)	N7^ii^—Mn3—N7—C24	6.0 (5)
Mn1—N1—C5—C6	−4.9 (7)	O2—Mn3—N8—C29	−85.7 (5)
C3—C4—C5—N1	2.8 (10)	O2^ii^—Mn3—N8—C29	80.9 (5)
C3—C4—C5—C6	−175.8 (6)	N8^ii^—Mn3—N8—C29	−179.6 (6)
C7—N2—C6—C5	−177.6 (6)	N7—Mn3—N8—C29	−4.2 (5)
Mn1—N2—C6—C5	7.7 (8)	N7^ii^—Mn3—N8—C29	168.4 (5)
N1—C5—C6—N2	−1.9 (9)	O2—Mn3—N8—C30	97.0 (7)
C4—C5—C6—N2	176.8 (6)	O2^ii^—Mn3—N8—C30	−96.4 (7)
C6—N2—C7—C8	−134.6 (7)	N8^ii^—Mn3—N8—C30	3.1 (6)
Mn1—N2—C7—C8	39.4 (9)	N7—Mn3—N8—C30	178.5 (7)
N2—C7—C8—C9	−72.3 (10)	N7^ii^—Mn3—N8—C30	−8.9 (10)
C7—C8—C9—N3	72.7 (10)	C28—N7—C24—C25	−0.3 (10)
C10—N3—C9—C8	137.2 (8)	Mn3—N7—C24—C25	−178.0 (5)
Mn1—N3—C9—C8	−40.1 (9)	N7—C24—C25—C26	0.4 (11)
C9—N3—C10—C11	178.3 (6)	C24—C25—C26—C27	−1.1 (11)
Mn1—N3—C10—C11	−4.1 (8)	C25—C26—C27—C28	1.6 (12)
C15—N4—C11—C12	1.0 (9)	C24—N7—C28—C27	0.8 (9)
Mn1—N4—C11—C12	179.7 (5)	Mn3—N7—C28—C27	178.9 (5)
C15—N4—C11—C10	−178.8 (6)	C24—N7—C28—C29	177.0 (6)
Mn1—N4—C11—C10	−0.1 (7)	Mn3—N7—C28—C29	−4.8 (7)
N3—C10—C11—N4	2.9 (9)	C26—C27—C28—N7	−1.4 (11)
N3—C10—C11—C12	−176.9 (6)	C26—C27—C28—C29	−177.5 (7)
N4—C11—C12—C13	−2.0 (11)	C30—N8—C29—C28	−179.2 (7)
C10—C11—C12—C13	177.8 (7)	Mn3—N8—C29—C28	3.2 (9)
C11—C12—C13—C14	1.9 (13)	N7—C28—C29—N8	1.2 (10)
C12—C13—C14—C15	−0.7 (12)	C27—C28—C29—N8	177.6 (6)
C11—N4—C15—C14	0.2 (11)	C29—N8—C30—C31	176.8 (7)
Mn1—N4—C15—C14	−178.4 (5)	Mn3—N8—C30—C31	−6.0 (11)
C13—C14—C15—N4	−0.3 (12)	N8—C30—C31—C30^ii^	3.2 (6)

Symmetry codes: (i) −*x*+1, *y*, −*z*; (ii) −*x*, *y*, −*z*+1.

### Hydrogen-bond geometry (Å, °)

**Table:**

*D*—H···*A*	*D*—H	H···*A*	*D*···*A*	*D*—H···*A*
O1—H1WA···Br2	0.932	2.34	3.261 (5)	170.1
O1—H1WB···Br2^iii^	0.850	2.64	3.268 (5)	131.6
O2—H2WA···Br4	0.913	2.23	3.145 (5)	175.2
O2—H2WB···Br3^iv^	1.037	2.21	3.234 (4)	169.5
C4—H4···O2^iii^	0.93	2.42	3.344 (9)	173
C29—H29···Br2^v^	0.93	2.88	3.742 (7)	154

Symmetry codes: (iii) −*x*+1/2, *y*+1/2, −*z*+1; (iv) *x*−1/2, *y*−1/2, *z*+1; (v) −*x*+1/2, *y*−1/2, −*z*+1.
